# Experience and Perceptions of a Family Health History Risk Assessment Tool among Multi-Ethnic Asian Breast Cancer Patients

**DOI:** 10.3390/jpm11101046

**Published:** 2021-10-19

**Authors:** Sungwon Yoon, Hendra Goh, Si Ming Fung, Shihui Tang, David Matchar, Geoffrey S. Ginsburg, Lori A. Orlando, Joanne Ngeow, Rebekah Ryanne Wu

**Affiliations:** 1 Health Services and Systems Research, Center for Population Health Research Institute, Duke-NUS Medical School, Singapore Health Services, 8 College Road, Singapore 169857, Singapore; sungwon.yoon@duke-nus.edu.sg; 2 Health Services and Systems Research, Duke-NUS Medical School, Singapore 169857, Singapore; hendra.goh@duke-nus.edu.sg (H.G.); sshtang@gmail.com (S.T.); david.matchar@duke-nus.edu.sg (D.M.); 3 Cancer Genetics Service, Division of Medical Oncology, National Cancer Centre Singapore, Singapore 169610, Singapore; simingfung@gmail.com (S.M.F.); joanne.ngeow.y.y@singhealth.com.sg (J.N.); 4 Department of Medicine, Duke University School of Medicine, Durham, NC 27708, USA; 5 Center for Applied Genomics and Precision Medicine, Department of Medicine, Duke University School of Medicine, Durham, NC 27708, USA; geoffrey.ginsburg@duke.edu (G.S.G.); orlan002@duke.edu (L.A.O.); 6 Lee Kong Chian School of Medicine, Nanyang Technological University, Singapore 308232, Singapore; 7 Center for Applied Genomics and Precision Medicine, Department of Medicine, Duke University School of Medicine, 304 Research Dr. Box 90141, Office 264, Durham, NC 27708, USA

**Keywords:** family health history, risk assessment, oncology, breast cancer, Asian, underrepresented populations

## Abstract

A family health history-based risk assessment is particularly valuable for guiding cancer screening and treatment strategies, yet an optimal implementation depends upon end-users’ values and needs. This is not only true prior to disease development, but also for those already affected. The aim of this study is to explore perceptions of the value of knowing one’s family health history (FHH)-based risk, experience using a patient-facing FHH tool and the potential of the tool for wider implementation. Twenty multi-ethnic Asian patients undergoing breast cancer treatment in Singapore completed an FHH-based risk assessment. Semi-structured one-on-one interviews were conducted and data were thematically analyzed. All participants were female and slightly more than half were Chinese. The acceptance and usage of an FHH risk assessment tool for cancers and its broader implementation was affected by a perceived importance of personal control over early detection, patient concerns of anxiety for themselves and their families due to risk results, concerns for genetic discrimination, adequacy of follow-up care plans and Asian cultural beliefs toward disease and dying. This study uniquely sheds light on the factors affecting Asian breast cancer patients’ perceptions about undergoing an FHH-based risk assessment, which should inform steps for a broader implementation in Asian healthcare systems.

## 1. Introduction

Family health history (FHH) is not only the strongest risk factor for rare diseases, but is well known to impact chronic disease risk as well [1,2]. An FHH-based risk assessment increases the recognition of disease risk and prompts discussion between high-risk family members and healthcare providers, facilitating the uptake of appropriate screening and preventive behaviors [3]. Despite the known benefits of an FHH-based risk assessment, widespread use remains limited by many factors [4], including patients’ inability to provide FHH due to poor communication among family members, providers’ lack of confidence in their genetic knowledge, and healthcare systems’ inadequate data standardization and support for data synthesis into risk-stratified prevention strategies [5,6,7].

In order to overcome some of these barriers, patient-facing FHH collection tools have been developed to improve risk stratification and disease prevention efforts [8,9,10]. These tools are designed to offer integrated clinical decision support (CDS) in the form of evidence-based recommendations to improve patient outcomes [11,12,13,14]. The use of these risk assessment platforms results in the improved risk stratification of populations and the uptake of guideline-driven care personalized to the individual.

A risk assessment is not only of value to patients prior to disease diagnosis, but is also critical for identifying hereditary syndromes in those already affected. The potential benefits are for both the individual and their family. For example, a cascade testing of healthy relatives to identify others at high risk for disease, and managing the already affected individual’s risk of developing additional diseases associated with the hereditary syndrome. In cancer hereditary syndromes, this frequently manifests as developing a second primary cancer, either of the same type (e.g., a primary breast cancer in each breast) or a different type (breast cancer and ovarian cancer in the same individual), which can occur sometimes several years later [15]. If these syndromes are identified at the first disease occurrence, measures can be put in place to actively monitor for second occurrences or prevent them through prophylactic surgeries or medications. In addition, with today’s targeted therapeutics, mutations associated with hereditary cancer syndromes can guide the selection of optimal cancer therapy—improving responsiveness while minimizing toxicity [16,17].

While the use of FHH-based risk assessment platforms has been well established in primary care environments, less has been performed to understand its utility in cancer clinics among individuals already affected. Additionally, the majority of these studies have been conducted in largely Caucasian populations. Given that implementation, particularly around genetic interventions, is very context specific, an evaluation of such interventions in diverse populations is imperative [18,19,20]. We performed a hybrid implementation effectiveness trial of an FHH-based risk assessment platform in breast oncology clinics in Singapore. The clinical utility of the intervention has been published [21]. In this paper, we describe the results of the qualitative analysis exploring breast cancer patients’ perceived value of knowing one’s genetic risks and their experience using the risk assessment platform. We also assess the perceived utility of implementing an FHH-based risk assessment in primary care in Singapore for a greater population impact.

## 2. Materials and Methods

### 2.1. Overview and Study Design

This was a hybrid type III implementation effectiveness trial conducted at two breast surgical oncology clinics in Singapore, a multi-ethnic city-state in Asia [22]. The study was approved by Duke University IRB (Pro 00087654) and SingHealth-Centralized Institutional Review Board (CIRB 2018/2046).

### 2.2. Participant Recruitment and Enrollment

Full details of the methods have been described elsewhere [21]. Briefly, patients were eligible if they were (1) diagnosed with a histologically confirmed breast cancer, (2) had not been referred for genetic counseling and/or testing previously and (3) were proficient in English. At study enrollment, patients were provided with information about the importance of FHH and an FHH worksheet listing relative categories with a description of the conditions collected in the platform. Patients were scheduled to enter relevant personal history and FHH information into the risk assessment platform before their next clinic appointment using an electronic device provided by the study team. Risk assessment reports were generated in real-time and provided to patients and providers. It was up to participants and their healthcare providers to discuss the results and decide whether to act on the recommendations for genetic counselling or surveillance.

For this qualitative sub-study, participants enrolled in the trial were asked to participate in a qualitative interview to explore the perceived value of risk assessment, the usability of the risk assessment platform, and the perceived benefit of broader implementation in primary care settings in Singapore. We used a purposive sampling technique to recruit participants at a range of ages, ethnicities and cancer stages to maximize the diversity of experiences and opinions.

### 2.3. Intervention

The risk assessment platform is a web-based, patient-facing platform, MeTree, that collects personal characteristics, medical history and family health history on 90 diseases and provides risk assessment and clinical decision support (CDS) for 30 based on commonly accepted guidelines for disease identification, prevention and surveillance [23]. MeTree has been evaluated in numerous healthcare systems in the United States and in Singapore [23,24,25]. It has been widely accepted in primary care populations and significantly increases the identification of individuals at risk for hereditary conditions [24,26,27].

### 2.4. Data Collection

A semi-structured interview guide was developed based on the study team’s expert knowledge and relevant literature [28,29]. The interview guide consisted of questions on the perceived benefits and risks of knowing one’s genetic risk and the importance of FHH-based risk assessment, experience of the MeTree tool and perceptions of the utility of wider implementation of a platform such as MeTree in primary care. Individuals consented verbally and in writing; then, completed a 30–45 min one-on-one interview with a trained qualitative researcher (ST). Reflections and field notes were documented after each interview to capture insights. To ensure thematic saturation (i.e., an additional interview did not yield any new themes), analyses were performed concurrently with data collection; interview recruitment continued until the point of information redundancy was reached.

### 2.5. Data Analysis

All interviews were audio-recorded and transcribed verbatim. We used grounded theory for thematic data analysis [30,31]. The grounded theory approach allowed emerging constructs and themes to emerge through iterations of data collection and analysis. Two coders (ST and HG) independently reviewed the interview materials, summarized and extracted meaningful statements and conducted open and axial coding using NVivo 12, a qualitative data analysis software [32]. During open coding, transcripts were analyzed to develop categories of information, which allowed subthemes to arise inductively from the data instead of from pre-existing ideas. During axial coding, common sub-themes were grouped into unifying themes. Themes and sub-themes were reviewed by both coders and any discrepancies were discussed and reconciled through several rounds of consensus meetings involving research team members. The iterative process continued until no new emergent themes were identified. For rigor and transparency, we anchored our methodology according to the Consolidated Criteria for Reporting Qualitative Research (COREQ) checklist [33].

## 3. Results

### 3.1. Characteristics of Participants

A total of twenty patients participated in one-to-one interviews. Data saturation was reached after the eighteenth interview, with no new themes emerging from subsequent interviews. We conducted two additional interviews beyond data saturation to ensure that the point of information redundancy was achieved. All participants were female with varying stages of breast cancer, nearly half (45%) were stage 2, and the majority were of Chinese ethnicity (55%). The mean age was 51.1 (±6.9) years old, with 85% falling between 40 and 59 years old. Mean years since cancer diagnosis was 4.4 (±2.1) years and 25% of the patients were referred to genetic counselling following the intervention (Table 1).

### 3.2. Perceptions of Knowing Genetic Risk of Cancer: Benefits and Harms

When asked about the benefits of knowing one’s genetic risk, many participants stated that understanding genetic risks would enable them to cultivate an internal locus of control to prepare for future adversity and, thus, respond to such situations with ‘peace of mind’.

*“Knowing the risks will make me more mentally prepared to handle the issues should they arise”. 43 Chinese, Stage 0.*

An FHH-based risk assessment was also viewed as prompting early financial planning to ensure family affordability for cancer treatment. As one participant mentioned,

*“Cost of cancer treatment can be exorbitant, and not many people can actually afford [it]. So, I think it’s good to have this preliminary assessment, so if they fall under this high-risk group, then they can go for a further test and be financially prepared if results show positive”. 43 Chinese, Stage 0.*

Some participants added that a genetic risk assessment would heighten the awareness of their family’s genetic risks which in turn could improve their health-seeking behaviors.

*“So, for example, I will be more health-conscious in a sense, do go for regular check-ups? I think this [knowing one’s risk] will be helpful”. 44 Chinese, Stage 1.*

Despite the perceived utility and value of the risk assessment by many, a few participants were more skeptical and raised some concerns. The most commonly noted hindrance was related to the emotional and psychological repercussions. Participants worried that information generated from an FHH-based risk assessment tool might cause undue distress for themselves and their families.

*“After I was diagnosed with cancer, I realized that some of my friends feel very emotional about it. Although knowing my risk is good, but it tends to give me the impression that people around me tend to get more emotional if the results are not ideal and I don’t want to face certain feelings”. 51 Malay, Stage 2.*

Given the absence of legal protections in Singapore at the time of data collection (Code of Practice for the provision of clinical genetic/genomic testing services has since been issued in December 2020 [34]; the code stipulates that all licensed genetic/genomic testing services are prohibited from disclosing genetic test results without prior consent to any third parties, including family members), some participants feared that they might face discrimination as the score obtained from the risk assessment could affect how others viewed them, ultimately limiting their social and employment opportunities.

*“Is it possible that the company you are applying for will get hold of such information? If so, then there is a risk of individuals facing employment discrimination”. 52 Chinese, Stage 2*.

### 3.3. Experience of MeTree: Usability

Although many participants reported being generally satisfied with their experience [21], there were mixed reactions to the usability of MeTree. Participants’ concerns could be categorized into three areas: *information entry, content and results documentation*. Participants generally liked the simple user interface of MeTree and its visual content, which they felt made it easy to complete the assessment.

*“I think the question with a pictorial representation would actually make a lot of sense to the user. Also, having a table like this makes it more comprehensible than the conventional way of presenting questions. Overall, it’s very simplified and easy to use”. 43 Chinese* *, Stage 1.*

However, some participants expressed frustration with specific aspects of the platform. The amount of information to be gathered and the overuse of medical terminology were the commonly mentioned hindrances reported by participants.

*“Because (there are) so many pages, like three pages, I didn’t go through every page. I only read the first page. Besides, those medical terms are not helping either”. 49 Indian, Stage 2.*

It is well established that patients need coaching on what and how to collect FHH information [35]. While the study provided worksheets for participants to collect FHH from family members prior to using the risk assessment platform, participants did not always do so [21]. Thus, some participants raised concerns about their inability to accurately input family members’ health history in their absence because some of the information was unfamiliar and hard to recall.

*“I realized I couldn’t complete the form when I was asked about my parents’ and relatives’ health condition[s]. It would be better if I can bring it back and discuss it with my family for a more accurate result”. 55 Chinese, Stage 2.*

Some participants experienced some challenges during the information entry due to a limited comprehension of the instructions provided by the MeTree platform, but appreciated the presence of the support staff, who helped with data entry and clarified concerns that emerged during the information entry process.

*“This [family genogram] is very hard to fill in. At least, the symbols and shapes on the genogram should be clearly stated. For example, square representing male and circle for female”. 65 Malay, Stage 3.*

*“Throughout the session, I needed guidance from the [research] coordinator. She helped to clear my doubts on ambiguous terms or questions. I need more instructions. I cannot do this at home alone”. 43 Chinese, Stage 0.*

Most participants liked the idea of being able to obtain their risk scores immediately upon submission of their data, which displayed their individualized risks based on their FHH. Participants also mentioned that having the results printed for their own record or for future discussion with a specialist was particularly valuable. There were cases where this was not possible or the records were misplaced. Participants particularly commented on the importance of maintaining that record.

*“Although the result is available immediately, I am not provided with the result for record-keeping purposes, which I can show to the doctor next time during the consultation”. 49 Others, Stage 2*.

### 3.4. Perceived Potential of MeTree for Wider Implementation

Many participants mentioned the importance of becoming aware of the genetic risks, yet we found two subtly different reactions to a wider implementation in primary care settings: (1) for “*others*” and (2) for their “*own family members*”.

For the recommendation for *others*, a recurrent theme was that the risk assessment could be beneficial for people, facilitating early detection and mitigating the risks of diseases that might present later. The following response from a participant was typical of many.

*“I think this [MeTree] is beneficial. I think everyone should go for it because five years ago, when I was diagnosed [with breast cancer], I did not fill [in] any of this questionnaire. So, I was actually thinking, “Could it be family history?” because my father was adopted, so we don’t know anything about his family history. I think this, it’s quite a good questionnaire so that people would know their family history, whether they are (considered) high risk, and if they are, they can go for further genetic tests”. 43 Chinese, Stage 0.*

However, a few participants were more skeptical about the implementation of a risk assessment in primary care and expressed reluctance. One of the reasons cited was the perceived high level of awareness and knowledge of cancer risk among the public in Singapore. Participants felt that in light of high-profile campaigns and the local media coverage of cancer, people become more aware of genetic and non-genetic determinants of cancer. Hence, the uptake of a risk assessment in primary care patients would be limited.

*“I don’t feel it really helped people in primary care. I think most Singaporeans already generally know if they are at risk. There are so many campaigns and programs. So, they will not use it [MeTree]” 45 Others, Stage 2*

Participants also pointed out cultural barriers that might hinder the uptake in primary care. As one participant stated, there are prevailing beliefs in Asian culture that discussion of future perils is often considered a taboo subject.

*“A lot of people are very sensitive. You can’t even mention the word “death” in front of them, not to mention asking them to take a survey that reveals their risk of developing diseases? That’s a big no-no”. 55 Chinese, Stage 2.*

Other participants were uncertain of the follow-up procedure upon the completion of the risk assessment. The perceived absence of systematic follow-up plans at a healthcare system level generated a sense of uncertainty, and these perceptions led to their hesitation to recommend the risk assessment to others.

*“So what can be expected after completing MeTree? The follow-up process is not made clear to us. Not so nice to leave us in a lurch. If resources allow, it will be nice to have a complete [follow-up] package. Otherwise, I don’t think people will find it helpful”. 52 Chinese, Stage 1.*

Similar to the theme for “others”, reasons cited for the recommendation to *family members* centered around the improvement of clinical outcomes as a result of early detection and diagnosis. It was believed that the completion of MeTree would prompt health-seeking behaviors among family members and empower them to make an informed decision.

*“I will definitely recommend it to my husband. Like my husband, whose father and mother have a lot of medical problems. So, once he knows his risk of developing diseases, he will take precautions to take care of his health”. 50 Chinese, Stage 4.*

However, many participants expressed concerns that the results of MeTree might create anxiety and felt conflicted as to whether one should share the genetic risks identified with other family members. To pre-empt these dilemmas, they expressed unwillingness to recommend the risk assessment to family members. As a patient articulated,

*“People are very sensitive. They don’t want to know. If you ask my husband, he wouldn’t want to know either. Instead of thinking about all these unhappy and uncertain things, he would rather not know anything. So, I don’t think I will recommend him for this genetic test so as not to generate more worries”. 55 Chinese, Stage 2.*

Some participants also questioned the reliability of the cancer risk assessment provided by MeTree, and this led to their reservation on recommending it to family members, if implemented in primary care. They questioned whether the likelihood of developing a condition in one’s lifetime could be determined by an FHH alone. Participants often mentioned their experience with close family members, highlighting the potential for MeTree or similar risk assessment tools to miss certain factors that contribute to disease risk.

*“I know the results will not be deterministic because every individual is different. If the person has a good lifestyle, maybe s/he won’t get cancer at all, right? Like me, I have seven sisters; three of us have (cancer), whereas the other four are perfectly fine. Do you get what I mean? Not hundred percent I will have cancer even if I’m high risk”. 43 Malay, Stage 4.*

## 4. Discussion

This qualitative study explored breast cancer patients’ perceptions toward knowing their FHH risks, the experience of using a web-based patient-facing FHH risk assessment tool and its perceived potential value in broader patient populations.

An individual’s likelihood to undergo an FHH-based risk assessment is often dependent on whether the assessment meets one’s outcome expectancy. Overall, participants in our study viewed the risk assessment as important for fostering personal control over early detection as a way to prepare for future adversity and financial planning for treatment. The risk assessment was also seen as a way to encourage preventive health behaviors. This was in line with previous literature that patients are motivated to undergo a genetic risk assessment to assert control over health outcomes [36,37]. Hence, clear communication of the benefits of the risk assessment in identifying risk and personalizing disease prevention and surveillance are important for wider implementation [38].

At the same time, participants expressed concerns about the potential for unnecessary anxiety for them and their family members as a result of the risk assessment. Multiple studies show that knowing familial susceptibility for cancer can engender a range of psychological responses, including increased anxiety and distress, which might hinder subsequent health-seeking behaviors [39,40]. These observations are particularly significant when promoting optimal prevention strategies for at-risk women. Exploring potential barriers during healthcare evaluations may alleviate patients’ concerns and enhance their acceptance of risk assessment recommendations [41]. Another important barrier that emerged was a concern of discrimination. Genetic discrimination is, as defined by the US National Human Genomic Research Institute, prejudice directed against people who have or may have genetic diseases which can involve denied employment or health insurance [42]. Globally, genetic discrimination remains one of the significant barriers preventing an adequate uptake of risk assessment as it is not uncommon for insurers or employers to obtain access to genetic information for underwriting purposes [43,44]. Concerns about genetic discrimination can be of particular public health importance in the context of advancing genetic technology and precision medicine because the fear of discrimination may prevent individuals from accepting genetic testing and counseling [45,46]. Thus, it is important to strengthen local policies that protect the privacy of health information while educating patients about existing protections against genetic discrimination.

Our study explored user experience, specifically the usability of MeTree as a potential risk assessment tool in Singapore. The use of a simple layout and visual content enhanced participant receptivity, while the length of the data input, unfamiliar medical terminology and limited understanding of the instructions hampered women from completing the tasks. It was also apparent that, for a minority of participants, navigating the MeTree platform independently was daunting. These findings suggest that additional modifications are likely necessary to improve the platform’s usability in the context of current oncology practice and beyond.

A novel finding from this study was that participants’ opinions regarding a wider implementation of MeTree or similar FHH-based risk assessment tools in primary care differed depending on the target recipients. For recommendations to “*others*”, the main benefit identified was early detection and the mitigation of risks, while key barriers were related to a perceived high level of awareness of genetic risks among the public, cultural taboos around the discussion of disease or death, and unclear follow-up care plans. This was consistent with prior literature demonstrating that follow-up procedures for high-risk patients remain suboptimal [47]. It will, therefore, be imperative to address these process gaps (e.g., nurse-led education to enhance genetic literacy and importance of surveillance), before attempting the widespread implementation of risk assessments [48,49].

For recommendations to “*family members*”, risk mitigation through early detection and preventive behaviors was the main perceived benefit, which was similar to the reasons espoused for recommendation to “*others*”. However, a major theme running through the interviews was an overall reluctance to recommend the risk assessment to family members, citing concerns about negative emotional ramifications and the impact on familial relationships. Intriguingly, a Dutch study reported an improved familial relationship upon the disclosure of genetic testing results. It brought families closer as a result of more frequent communication, improved support systems, and the obligation to ride out the storm together [50]. Similarly, a trial in the United States showed that use of an FHH-based risk assessment increased communication with family members, particularly among those with an increased risk for a greater number of conditions [3]. Asian cultural values, such as not wanting to trouble or pose inconveniences to others, may account for the difference in sentiments and actions observed between Caucasian and Asian patients [51]. Nonetheless, strategies to address complex familial dynamics after revealing genetic results may help improve the acceptance of a risk assessment, especially in an Asian context. One such strategy would be to integrate psychological support such as counseling into the care pathway, focusing on disease preventability to foster communication within the family [52].

This study had several limitations. Participants were patients being actively treated for breast cancer in an oncology setting and, therefore, findings may not be generalizable to other clinical settings or conditions. They may not be generalizable to those who were previously treated for breast cancer, as active cancer treatment comes with an enormous emotional and psychological burden that may significantly influence perceptions. However, the interview topics covered were sufficiently broad to have potential applicability to other potentially heritable health conditions. There was also the potential for selection bias, where women with a greater interest in expressing their opinions about MeTree may have been more likely to participate, which could have affected the findings of this study. Additionally, there was a time lapse of a week to 18 months between the administration of MeTree and qualitative interviews, owing to patients’ personal commitments, competing health priorities and psychological issues. This might have engendered the recall bias when assessing specific aspects of MeTree. Lastly, patients’ perceptions might have been affected by the time elapsed since diagnosis; it is possible that some patients who had survived long-term, had already learned of genetic risks through their own education and research prior to MeTree administration and, hence, saw the tool as less useful.

This study adds important empirical evidence to the literature by understanding the user experience of a risk assessment platform among multi-ethnic patients in an Asian oncology setting and their perceptions of the potential impact of a wider implementation in primary care in Asia. Most studies in Asian populations were still within Western societies and often were conducted among patients not currently affected by the disease [40]. Furthermore, they rarely involved the testing of an actual risk assessment platform, but were more theoretical discussions of risk and genetics. Our findings provide valuable insight into the future improvement and adaptation of FHH-based risk assessment platforms through modifications tailored to users’ needs and addressing barriers to implementation and acceptance.

## 5. Conclusions

With the potential health impact of a systematic risk assessment becoming increasingly evident, the need for access to such services is more apparent than ever [11,27,53]. However, access alone is insufficient to ensure an optimal patient uptake. Our findings suggest that acceptance and the usage of an FHH-based risk assessment tool and its wider implementation may be affected by the perceived importance of personal control over early detection, potential of risk results to generate anxieties for patients and their families, perception of genetic discrimination, adequacy of follow-up care plans and cultural beliefs toward disease and dying. These findings will inform future implementation strategies to improve the uptake of a systematic risk assessment and a wider implementation in Asian societies.

## Figures and Tables

**Table 1:** Demographic characteristics of participants (*n* = 20).

**Characteristics**	***N* (%)**
**Age**	51.1 ± 6.9
40–49	9 (45.0)
50–59	8 (40.0)
60–69	3 (15.0)
**Ethnicity**	
Chinese	11 (55.0)
Malay	3 (15.0)
Indian	1 (5.0)
Others	5 (25.0)
**Breast Cancer Stage**	
0	1 (5.0)
1	3 (15.0)
2	9 (45.0)
3	1 (5.0)
4	6 (30.0)
**Mean Years since Cancer Diagnosis**	4.4 ± 2.1
**Referred for Genetic Counselling**	
Yes	5 (25.0)
No	15 (75.0)

## Data Availability

Data from this study will be made available upon request from the authors.
